# Pandemic response policies’ democratizing effects on online learning

**DOI:** 10.1073/pnas.2026725118

**Published:** 2021-03-11

**Authors:** Rene F. Kizilcec, Christos A. Makridis, Katharine C. Sadowski

**Affiliations:** ^a^Department of Information Science, Cornell University, Ithaca, NY 14853;; ^b^W. P. Carey School of Business, Arizona State University, Tempe, AZ 85287;; ^c^Sloan School of Management, Massachusetts Institute of Technology, Cambridge, MA 02142;; ^d^Department of Policy Analysis and Management, Cornell University, Ithaca, NY 14853

**Keywords:** online learning, equity, future of work, COVID-19

## Abstract

The COVID-19 pandemic has changed peoples’ lives in unexpected ways, especially how they allocate their time between work and other activities. Demand for online learning has surged during a period of mass layoffs and transition to remote work and schooling. Can this uptake in online learning help close longstanding skills gaps in the US workforce in a sustainable and equitable manner? We answer this question by analyzing individual engagement data of DataCamp users between October 2019 and September 2020 (*n* = 277,425). Exploiting the staggered adoption of actions to mitigate the spread of COVID-19 across states, we identify the causal effect at the neighborhood level. The adoption of nonessential business closures led to a 38% increase in new users and a 6% increase in engagement among existing users. We find that these increases are proportional across higher- and lower-income neighborhoods and neighborhoods with a high or low share of Black residents. This demonstrates the potential for online platforms to democratize access to knowledge and skills that are in high demand, which supports job security and facilitates social mobility.

## Significance

In the face of rising university tuition costs and a longstanding skills gap in the US workforce, a growing number of people access higher and continuing education programs via online platforms. There are serious concerns that online learning disadvantages members of underserved communities, thereby exacerbating social inequalities. However, it is hard to evaluate these concerns at scale partly due to selection effects. Policy responses to the COVID-19 pandemic, such as nonessential business closures, suddenly changed how people spent their time, which allowed us to estimate effects on demand for online learning and how it varies along socioeconomic dimensions. Unlike most prior studies that find education technology to maintain or amplify inequities, we present causal evidence for its potential democratizing effects.

Productivity growth in the digital economy has expanded at roughly four times the rate of the overall economy, raising the demand for skilled knowledge workers (1, 2). As the cost of university tuition keeps rising, educational technology (EdTech) companies have moved into the education marketplace to meet specific industry demands at lower cost (3). Initial expectations that affordable online programs would have a democratizing effect by broadening educational access were met with the sobering reality that course takers tended to be highly educated (4) from high-income zip codes in the United States (5) and highly developed countries worldwide (6, 7). These findings have raised doubts among individuals and policy makers about the extent to which online learning can provide a much-needed boost in skills training for historically underserved populations.

The ongoing coronavirus pandemic has accelerated these patterns, reducing the returns of a traditional college education after the transition away from in-person classes (8). Students and working professionals have responded in part by substituting toward online learning with EdTech platforms. State policies such as stay-at-home orders (SAHOs) and nonessential business closures (NBCs) have compelled people to stay at home (9, 10). While many high-skilled jobs transitioned to remote work, lower-skilled jobs were terminated or furloughed at an unprecedented rate (11, 12).

This sudden shock led people to shift their allocation of time, including a substantial increase in investments in human capital accumulation. A leading online course provider, Coursera, saw a jump in its visitor numbers from 27 million to over 70 million between February and July 2020 (13). The increased interest was concentrated in computer science and programming courses that have been widely promoted as stepping stones to high-skilled career paths (14). The sudden and drastic changes to people’s lives due to the pandemic can offer answers to the question about how online learning might support efforts to upskill and retrain vulnerable segments of the workforce.

This research investigates the pandemic's effect on the adoption and use of online learning and its implications for the future of education in the years ahead. Drawing on individual-level records from DataCamp (15), one of the largest online learning platforms tailored toward programming skills, we use variation in the time that different US states adopted NBCs to estimate effects on the demand for online learning. In other words, we determine whether NBCs caused an increase in DataCamp usership and/or an increase in engagement among existing users by comparing rates across and within states over time.

We exploit the staggered adoption of NBC across states because it provides plausibly exogenous (i.e., unconfounded) variation in the time that individuals allocate between employment and personal activities. Our identifying assumption is that unobserved shocks to the demand for online learning are uncorrelated with the adoption of NBC. Since these state policies are driven much more by political affiliation than by the number of local infections or other real factors (16), we argue that these unobserved shocks are likely uncorrelated with a state’s decision to adopt an NBC. We nonetheless control for potential confounders such as the number of infections per capita and social distancing compliance.

Our results indicate that the passage of NBC is associated with a 38% increase in new DataCamp users and a 6% increase in engagement among existing users. This suggests that the pandemic brought people to start acquiring programming skills online (intensive margin effect) as well as it encouraged already active learners to study more (extensive margin effect). We observe little variation between lower- and higher-income regions and no variation based on racial composition. All demographic regions experienced a substantial increase in online learning following the adoption of these state policies. This suggests that, at least over the pandemic, the expansion of online learning through DataCamp has had a “democratizing” effect in the market for technical (programming) educational services.

## Results

Our samples of new and existing users (*n* = 277,425 and *n* = 69,942, respectively) across (*n* = 13,617 and *n* = 8,776, respectively) zip code-based regions resemble the broader US population along several key dimensions (*SI Appendix*, Table S1). Although the regions represented in our samples are more populated (mean = 9.59 and 9.93 individuals [log transformed], SD = 0.92 and 1.09, respectively) compared with all US regions (mean = 7.93, SD = 1.86), the median household income, percentage of college-educated individuals, and percentage of Black residents are only marginally higher than the population average. Moreover, the share of individuals in the retail trade, wholesale trade, and information services in our samples is not significantly different from the population. Our sample therefore adequately represents regions that were particularly hard hit by the pandemic: poorer and racially diverse regions with lower education levels and less digitally intensive workers (17).

**Fig. 1.**
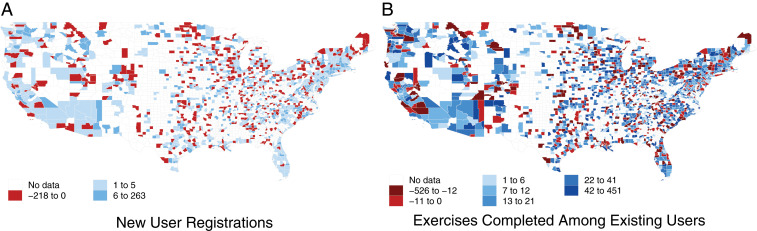
County-level change in online learning demand from May 2019 to May 2020. The county-level maps report growth in new user registrants divided by the county population and multiplied by 100,000 to get the rate per 100,000 residents (*A*) and the growth in the average number of exercises completed per user (*B*).

We observe a strong increase in the demand for online learning after the start of the pandemic. In May 2020, the first month that every state had implemented NBC, there were 25,395 new user registrations on DataCamp, up 59% from 15,982 in May 2019 and 15,385 in May 2018. Likewise, the average number of weekly exercises individuals completed was 37.8 in May 2020 compared with just 28.8 in May 2019 and 28.7 in May 2018. These trends in demand are shared across most parts of the United States, as visualized in Fig. 1.

Using the staggered implementation of NBC as a causal identification strategy, we arrive at a set of causal effect estimates. Tables 1 and 2 present five models for each outcome measure: the number of new user registrations and the number of exercises completed by existing users, respectively. Starting with a raw correlation coefficient with NBC, we progressively add more control variables and fixed effects to the model to mitigate concerns of omitted variables bias. Adjusting for the zip code employment distribution, for example, accounts for the possibility that areas with higher shares of retail employment are more adversely affected by the pandemic and could see especially high demand for online learning for reskilling and career change.

**Table 1.** Average treatment effects of NBC on demand for online learning in terms of the number of new user registrations

	Log(new user registrations)				
	(1)	(2)	(3)	(4)	(5)
NBCs	0.09***	0.14***	0.37***	0.37***	0.38***
	[0.01]	[0.01]	[0.02]	[0.02]	[0.02]
Zip code: log(total population)		0.14***	0.14***	0.16***	0.16***
		[0.01]	[0.01]	[0.01]	[0.01]
Zip code: log(median household income)		0.02	0.02	0.05	0.05
		[0.03]	[0.03]	[0.03]	[0.05]
Zip code: White, %		−0.25***	−0.25***	−0.37***	−0.45***
		[0.04]	[0.04]	[0.04]	[0.08]
Zip code: Black, %		−0.13***	−0.12***	−0.24***	−0.36***
		[0.04]	[0.04]	[0.04]	[0.06]
Zip code: under 18 years old, %		−0.48***	−0.48***	−0.63***	−0.75***
		[0.12]	[0.12]	[0.12]	[0.13]
Zip code: 18 to 24 years old, %		0.53***	0.51***	0.52***	0.45***
		[0.08]	[0.08]	[0.10]	[0.11]
Zip code: 25 to 44 years old, %		0.56***	0.56***	0.58***	0.51***
		[0.06]	[0.05]	[0.06]	[0.07]
Zip code: college or more, %		0.51***	0.52***	0.60***	0.57***
		[0.06]	[0.06]	[0.06]	[0.05]
Zip code: poverty White, %		0.26***	0.27***	0.30**	0.33*
		[0.08]	[0.08]	[0.11]	[0.17]
Zip code: poverty Black, %		0.07**	0.06**	0.08**	0.10**
		[0.03]	[0.03]	[0.04]	[0.04]
Zip code: industry retail, %		−0.57*	−0.55*	−0.50	−0.61**
		[0.29]	[0.28]	[0.30]	[0.29]
County: cases per capita				6.72***	6.52***
				[1.20]	[1.19]
County: social distancing compliance				0.47***	0.46***
				[0.03]	[0.03]
*R*^2^	0.01	0.14	0.17	0.24	0.25
Sample size	164,574	153,410	153,410	109,555	109,555
Week fixed effect	No	No	Yes	Yes	Yes
State fixed effect	No	No	No	No	Yes

The table reports the coefficients associated with regressions of logged weekly new user registrations on an indicator for whether the state has an NBC in place, conditional on controls and state and week fixed effects. Zip code controls include logged total population, logged median household income, the race distribution (the share White, Black), the age distribution (the share under age 18, 18 to 24, 25 to 44), the share of individuals with at least a college degree, the poverty rate for Whites and Blacks, and the share of workers in the retail trade sector. County controls include cumulative coronavirus cases per capita and social distancing compliance from Unacast. Significance is denoted with asterisks: *P < 0.10; **P < 0.05; ***P < 0.01. Sources are DataCamp (15), American Community Survey 2014 to 2018 (29), and Unacast (31).

**Table 2.** Average treatment effects of NBC on demand for online learning in terms of the number of exercises completed by existing users

	Log(exercises completed by existing users)				
	(1)	(2)	(3)	(4)	(5)
NBCs	0.05***	0.05***	0.11***	0.10***	0.06*
	[0.01]	[0.01]	[0.03]	[0.03]	[0.03]
Zip code: log(total population)		0.04***	0.04***	0.05***	0.05***
		[0.01]	[0.01]	[0.01]	[0.01]
Zip code: log(median household income)		−0.02	−0.02	−0.03	0.00
		[0.05]	[0.05]	[0.04]	[0.05]
Zip code: White, %		0.11**	0.11**	0.02	0.08
		[0.04]	[0.04]	[0.07]	[0.09]
Zip code: Black, %		0.08	0.07	0.04	0.11
		[0.07]	[0.07]	[0.08]	[0.08]
Zip code: under 18 years old, %		−0.25	−0.24	−0.33	−0.39
		[0.23]	[0.23]	[0.25]	[0.29]
Zip code: 18–24 years old, %		0.26**	0.25**	0.09	0.09
		[0.12]	[0.12]	[0.13]	[0.12]
Zip code: 25–44 years old, %		0.01	0.01	−0.20**	−0.21**
		[0.10]	[0.09]	[0.09]	[0.09]
Zip code: college or more, %		−0.01	0.00	0.05	−0.01
		[0.06]	[0.06]	[0.05]	[0.05]
Zip code: poverty White, %		−0.19	−0.19	−0.25	−0.2
		[0.18]	[0.18]	[0.21]	[0.22]
Zip code: poverty Black, %		0.01	0.01	0.06	0.06
		[0.06]	[0.06]	[0.07]	[0.07]
Zip code: industry retail, %		−0.55*	−0.54	−0.39	−0.59
		[0.33]	[0.33]	[0.41]	[0.47]
County: cases per capita				3.42***	6.16***
				[0.86]	[1.53]
County: social distancing compliance				0.01	−0.01
				[0.05]	[0.05]
*R*^2^	0.00	0.00	0.00	0.00	0.00
Sample size	295,546	263,764	263,764	124,089	124,089
Week fixed effect	No	No	Yes	Yes	Yes
State fixed effect	No	No	No	No	Yes

The table reports the coefficients associated with regressions of weekly exercises completed among existing users on an indicator for whether the state has an NBC in place, conditional on controls and state and week fixed effects. Zip code controls include logged total population, logged median household income, the race distribution (the share White, Black), the age distribution (the share under age 18, 18 to 24, 25 to 44), the share of individuals with at least a college degree, the poverty rate for Whites and Blacks, and the share of workers in the retail trade sector. County controls include cumulative coronavirus cases per capita and social distancing compliance from Unacast. Significance is denoted with asterisks: *P < 0.10; **P < 0.05; ***P < 0.01. Sources are DataCamp (15), American Community Survey 2014 to 2018 (29), and Unacast (31).

For new user registrations, we find that the introduction of NBC is associated with a 9% rise in new users before adding any controls (column 1). The effect estimate rises, rather than falls, to 14% after adding zip code-specific demographic characteristics (column 2), which suggests that the areas that were harder hit by the pandemic were more likely to experience an increase in new users. The effect estimate further increases to 37% after we control for week fixed effects (column 3), which eliminates variation from the fluctuations in aggregate demand for online learning that could be correlated with state pandemic responses.

However, these estimates could still be biased due to the combination of time-varying or time-invariant state characteristics that are correlated with NBC adoption. To address these concerns, we control for the weekly number of coronavirus cases per capita and an index of compliance with social distancing policies (column 4). Our coefficient estimate on NBC remains the same, suggesting that our results do not reflect reverse causality stemming from the rise in the severity of coronavirus or other social distancing policies. Finally, we also add state fixed effects to observationally compare online learning interactions before vs. after the adoption of the NBC (column 5). Given that our coefficient estimates are statistically indistinguishable with and without state fixed effects, we conclude that selection effects—the possibility that individuals in some states are simply more likely to start online learning than others—play a limited role in accounting for the surge in online learning.

For weekly exercises among existing users, we find a similar pattern of results. In particular, the raw correlation indicates that individuals in states that adopted NBC have 5% higher weekly engagement (column 1). The inclusion of zip code demographic characteristics does not alter the effect estimate (column 2). After we introduce week fixed effects, the estimate rises to 11% (column 3), consistent with the direction of bias present for the user registration outcome. Further adjusting for weekly coronavirus cases per capita and compliance with social distancing policies produces a statistically indistinguishable estimate (column 4). Finally, when we exploit within-state variation, we find that the adoption of NBC is associated with a 6% rise in weekly engagement (column 5), which is significant at the 10% level. Although the effect magnitude and statistical significance decline, we still find robust evidence that NBC led to increased adoption of online learning and allocation of time to online learning activities.

**Fig. 2.**
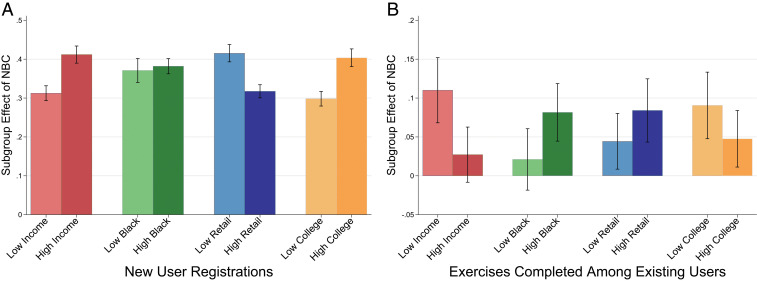
Heterogeneity in the effects of NBC on new user growth (*A*) and engagement among existing users (*B*). Subgroup effect estimates were derived using regression specification (5) in Tables 1 and 2. Sociodemographic subgroups are defined by whether an individual resides in a zip code below (low) or above (high) the US median for each characteristic. Error bars indicate cluster-robust SEs.

Having examined average treatment effects, we next investigate sociodemographic heterogeneity and the effect of NBC on online learning. Specifically, we define subgroups in terms of household income and the share of residents who are Black, retail workers, and college educated. Fig. 2*A* presents evidence that the effect on new user registrations is statistically significantly larger in higher-income and more educated regions and lower in regions with higher employment shares in the retail sector. This finding is consistent with the expectation that economic returns to programming skills are larger in professional services jobs, which is positively correlated with education and negatively correlated with retail employment. However, the effect sizes are statistically indistinguishable between regions with a high vs. low share of Black residents, suggesting that minority communities had equal access to online learning. In sum, the adoption of NBCs is associated with a 30 to 40% increase in new registrations across demographically diverse regions, and effect heterogeneity favors more affluent and educated areas by only about 10 percentage points (*SI Appendix*, Table S2 has detailed estimates).

We find further evidence of a democratizing effect in terms of existing user engagement, shown in Fig. 2*B*. Specifically, we find treatment effects that are nearly twice as large for the number of weekly exercises completed among individuals in regions with below the median household income, above the median share of Black residents, above the median share of retail employment, and below the median share of college graduates. Although the CIs on these treatment effects are larger and we cannot rule out that the treatment effects are the same within a 95% CI with the exception of the results for high- vs. low-income regions, overall these results suggest that online learning platforms like DataCamp can be an important tool for facilitating upskilling among workers in lower-skilled areas (*SI Appendix*, Table S3 has detailed estimates).

## Discussion

The coronavirus pandemic has been associated with a wide array of negative outcomes, ranging from mass unemployment to elevated suicide risk (18). This research focuses on a positive outcome: how the disruption has affected people’s time allocation between market activities and human capital investments. The severity of the pandemic eventually led all states to implement mitigation policies, which we find to have increased demand for online learning. Specifically, we observe more people signing up to learn coding skills and more engagement among existing users following the implementation of NBCs. These findings suggest that individuals are willing to make human capital investments, but they potentially perceive not having the time to undertake these investments when they are working full time. Behavioral science interventions that facilitate strategic plan making and time management could therefore help workers achieve their professional learning goals (19, 20).

The effects of the pandemic have been more severe in disadvantaged communities, which also tend to benefit less from advances in EdTechs (21). For example, massive open online courses have seen lower adoption and success rates in lower-income regions (5, 6). In contrast, we find that online learning engagement among existing users rose significantly in lower-income regions but not in higher-income regions. Adoption from new users was consistently large, with somewhat higher rates in higher-income and more educated regions.

Taken together, the results provide evidence that online platforms can provide an accessible and inclusive environment to learn new skills. Although we cannot say how much different people benefit from online learning, our results reveal trends among people in different zip codes. As a proxy for individual differences, zip code-level demographics tend to exaggerate trends by combining individual and community characteristics (22), but these discrepancies are larger for small, rural areas than for large, urban areas (23). Our sample tends to be from larger urban areas (*SI Appendix*, Table S1), as DataCamp targets young professionals and university students, thus mitigating zip code-based discrepancies. Prior work showing sociodemographic inequalities in online courses has also been conducted at the zip code level and even national level (5, 6). Despite the lack of individual-level characteristics, our current findings therefore provide evidence consistent with the idea that online learning can have democratizing effects.

With all the business closures over the pandemic (12), including childcare centers (9), women have been especially hard hit as parental responsibilities have fallen more on them (24). How NBCs affected women’s demand for online learning compared with men’s is especially interesting given that technology firms have been critiqued for being a male-dominated industry. A limitation of not having individual-level demographic data is our inability to speak to gender differences and address engagement among women and parents. Nevertheless, we do demonstrate that regions with higher proportions of individuals aged 25 to 44 have increasingly joined DataCamp and existing users in those regions increased their learning engagement. This offers suggestive evidence that the pandemic eased time constraints on education, at least for some, and thereby, led to behavior change with regard to seeking out learning opportunities.

This study examines a relatively short time period and is not linked to user reports of career advancement or hiring success. We therefore cannot draw conclusions about whether the increased demand and engagement in online learning have stimulated career advancements. An impact report from a similar online learning platform, Coursera, surveyed over 51,000 users and found 72% reported career benefits from taking online courses (25). These benefits included a small fraction receiving a promotion and about half of the respondents saying that they now have better credentials for future jobs. While Coursera offers a wider variety of courses than DataCamp, the findings are promising and suggest DataCamp users who grew their skills over this time frame could also benefit professionally. This is especially applicable in the context of DataCamp, which serves a user base of firms and technology teams with demand for upskilling their employees. Our focus on programming courses in this study leaves room for future research on the impact of online upskilling in other industries, such as management and advanced manufacturing, where training tends to involve more social and hands-on learning experiences.

The coronavirus pandemic and ensuing mitigation policies have changed people’s lives in many ways. The forced closure of businesses and directives to stay socially isolated increased the amount of “free time” for some individuals by reducing commute times as well as time spent on social activities. For others, such as women and parents, this newly found free time was filled with new and greater responsibilities such as homeschooling and child care. While demands on time increased for some, our data suggest that at least a portion of the US population benefited from new found free time and used that time to upskill. Furthermore, it appears that this occurred relatively consistently across regions with varying income, racial, educational, and occupational compositions. These findings suggest an interesting set of interventions for businesses and policy makers to consider. The closure of nonessential businesses acted as a large shock to free time for some individuals; if businesses could increment the time off they give to their employees, they could potentially support individuals in the pursuit of career development goals. This may provide a sustainable and equitable approach toward closing the longstanding skills gap in the US workforce.

## Materials and Methods

### Study Design.

We study the demand for online learning using records from DataCamp (15), one of the fastest-growing online learning platforms for programming and data science courses. The platform offers nearly 400 courses on Python for data science, R programming, SQL databases, and applied finance. The courses are self-paced and interactive. The first “chapter” of any course is provided for free, and additional materials are offered for a monthly fee (between US$25 and $33 per person for individual and business plans). DataCamp’s user base is composed primarily of professionals who would otherwise be thinking of going back to formal school for more training, employees who have a good experience with DataCamp and then advocate to incorporate the platform into their teams at work, and professors at universities who use the DataCamp platform to teach their students new skills. DataCamp serves 80% of Fortune 1000 companies, helping them upskill their employees. While it is not as large as massive open online course (MOOC) providers like Coursera or edX, DataCamp is recognized as one of the fastest-growing technology, media, telecommunications, life sciences, and energy tech companies in North America, with a total of 618,000 active learners (26).

We obtained deidentified individual-level data from DataCamp between January 2018 and September 2020 with information on which courses individuals have taken, which exercises they completed, whether they completed the course, their platform subscript status, and their zip code-level location. Our sample consists of 618,000 users who are located in the United States and who have completed at least one course exercise. Depending on the target outcome in the analysis, we narrow down our focal sample to either 277,425 users who newly registered or 69,942 users who were active on the platform during the 22 wk before or after NBCs went into effect. We chose 22 wk because the last week in our sample where all 50 states and the District of Columbia were represented was 22 wk after NBC. We draw on the COVID-19 State Policy Database for an official timeline of state policies such as NBC and SAHOs (27, 28).

Since DataCamp does not collect individual demographics, we use zip code characteristics as a proxy from the Census Bureau's 5-y American Community Survey from 2014 to 2018 (29). While geographic aggregations are an imperfect proxy for individual characteristics (30), our focus is on comparing the response of learners in different zip codes, but the same state, before vs. after the adoption of NBC. Since zip codes are much more disaggregated than our state-level variable of interest (NBC), measurement error is unlikely to create bias.

For each individual’s zip code, we observe the population density, the median household income, the income distribution (the share with less than $15,000, $15,000 to $29,000, $30,000 to $39,000, $40,000 to $49,000, $50,000 to $59,000, $60,000 to $99,000, $100,000 to $149,000, over $150,000), the race distribution, the age distribution (the share under age 18, age 18 to 24, age 25 to 44, age 45 to 64, and age 65+), the education distribution (the share with less than a high school degree, some college, and college or more), the poverty rate (the share of people living in poverty under age 18, age 18 to 64, and age 65+), and the industry and occupational distribution. We also recognize that, while there might be heterogeneity in how male and female users respond to NBC, there is not enough variation across zip codes in the share of males vs. females to tease out these effects. *SI Appendix*, Table S1 compares the demographic characteristics of zip codes in our sample with those in the entire United States.

Finally, we obtain daily county data on coronavirus cases and deaths from the Johns Hopkins Coronavirus Resource Center and weekly county data on compliance with social distancing policies from Unacast (31). Their social distancing scores are based on cell phone global positioning system (GPS) data, which they use to compute the average distance traveled per device for each day and the average distance traveled on the same weekday during the 4 wk prior to the pandemic between 10 February and 8 March 2020. By comparing distance traveled each day with the average prepandemic, they provide a measure of compliance that normalizes for differences across space in travel activity. We report summary statistics to compare our samples of new and existing users with the broader US population along several key dimensions at the zip code and county levels in *SI Appendix*, Table S1.

### Outcome Measures.

We focus our analysis on two different outcomes to distinguish between an effect on people deciding to start online learning (a measure of the extensive margin in online learning) and an effect on the level of engagement among existing online learners (a measure of the intensive margin). The first outcome measure is defined by the number of platform registrations per week. The second outcome measure is defined by the sum of exercises completed per week by existing users. Existing users are defined as having registered at least 22 wk prior to the start of the NBC in their state and as having completed at least one exercise during the 44-wk window surrounding the NBC. Both outcome measures are aggregated to the zip code level by summing over individuals.

### Covariate Measures.

We use a number of covariate measures for regression adjustment and to estimate heterogeneous treatment effects. Since we do not have individual demographic characteristics, we use zip code characteristics as a proxy. For each zip code, we consider the following characteristics as covariates: total population (log transformed), median household income (log transformed), race distribution (percentage of White, Black individuals), age distribution (percentage under the age of 18, 18 to 24, 25 to 44), the percentage of individuals with at least a college degree, the poverty rate among White and Black individuals, and the percentage of workers in the retail trade sector.

Additionally, we use two time-varying county-level variables for regression adjustment: the number of cumulative coronavirus cases per capita and a measure of social distancing compliance from Unacast. These measures help control for changes in the intensity of the pandemic and the adherence to the policies that could otherwise explain differences in the demand for online learning. For example, areas that were more adversely affected could experience an exodus of residents; controlling for these factors ensures that the comparison is between observationally equivalent areas.

To analyze heterogeneity in the average treatment effects, we created four indicator variables to identify relatively more vulnerable populations in terms of income, race, employment sector, and level of education. Specifically, the indicators record if a user lives in a zip code with above median 1) log-transformed median household income, 2) share of the population that is Black, 3) share of workers in the retail trade sector, and 4) share of the population with a college or higher degree.

### Statistical Analysis.

**Fig. 3.**
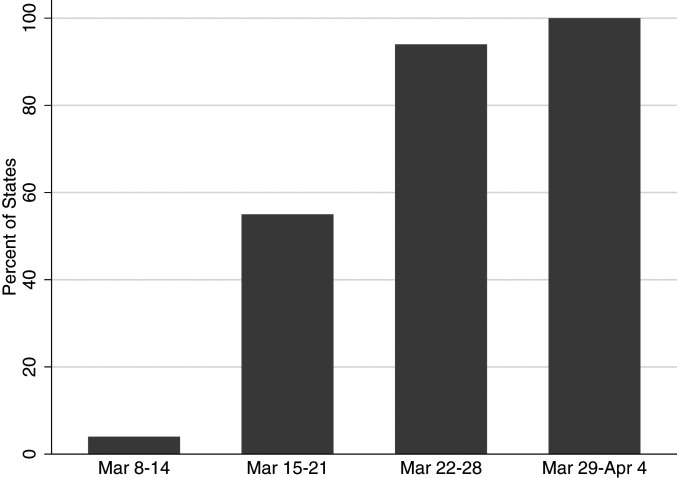
Percentage of states with NBCs between 8 March and 4 April 2020.

Our analytic strategy is to use the fact that NBCs were implemented in some states sooner than others. Our identifying assumption is that unobserved shocks to the demand for online learning are uncorrelated with the time that states enacted their NBC, conditional on a set of observable covariates including coronavirus cases. Because states adopted NBC policies at different points in time, state fixed effects allow us to compare outcomes in states that adopted NBCs sooner with those that adopted them later. Fig. 3 shows the share of states with NBC over time: it increases from 4 to 100% between the second and fifth weeks of March 2020. This staggered adoption of NBC provides sufficient temporal variation to identify causal effects. We decided to focus on NBC rather than SAHO in our analysis partly because SAHOs were implemented within a more compressed period of time, providing less within-state variation.

We acknowledge that the pandemic affected a range of industries asynchronously and led to sporadic closures across sectors by states, which make a perfect identification strategy difficult. To ensure our decision was meaningful, we studied two of the major policies by state, the SAHO and NBC. SAHO generally occurred 1 or 2 wk after NBC. We found that the change in our outcomes aligned more closely with NBC, namely that the increases in users and exercises aligned with this date for our sample. This makes intuitive sense as we consider the nature of our project, exploring change in online learning, which is likely associated more with individuals who would be affected by NBC.

Our identification strategy yields robust causal estimates as long as unobserved determinants of the decision to start online learning with DataCamp are uncorrelated with the timing of NBC. A recent study showed that the timing and type of state policies that were adopted were strongly correlated with political affiliation, which is unlikely to be correlated with the demand for online learning (16). Still, we use a combination of fixed effects models and regression adjustment to mitigate potential sources of confounding.

We use the following regression specification to estimate causal effect of NBC on the demand for online learning:

$$y_{izt}=\gamma P_{st}+g\left(X_{zt},\theta \right)+\phi_{s}+\lambda_{t}+{\mathrm{Ò\epsilon}}_{izt},$$

where *y*_*ilt*_ denotes our measure of online learning (e.g., logged new registrations or exercises) for individual *i* in zip code *z* and time *t*, *P*_*st*_ denotes an indicator for whether an NBC was in effect in state *s* at time *t*, *g*(*X*_*zt*_, θ) denotes a semiparametric function of demographic controls at the location level, and $\phi_{s}$ and $\lambda_{t}$ denote fixed effects on state and time, respectively. All time-varying measures are aggregated at the weekly level to smooth out cyclical variation within each week. SEs are clustered at the state level to allow for autocorrelation over time in the same geography.

## Supplementary Material

Supplementary File

## Data Availability

Data analysis scripts and some aggregate data are available at Open Science Framework (https://osf.io/45qxb/). Requests for the full dataset need to be directed to DataCamp (https://www.datacamp.com/).
