# Supplementary material for: Pandemic response policies’ democratizing effects on online learning
Source: Proc Natl Acad Sci U S A. 2021 Mar 11;118(11):e2026725118. doi: 10.1073/pnas.2026725118 (PMC7980394; doi:10.1073/pnas.2026725118)
Supplement: Supplementary File [file pnas.2026725118.sapp.pdf]

PNAS
www.pnas.org

Supporting Information for

Pandemic Response Policies' Democratizing Effects on Online Learning

This PDF file includes:

Table S1
Table S2
Table S3
References

Table S1. Demographics characteristics. Summary statistics of demographic characteristics for the U.S. population, new user sample, and existing user sample.

	(1) U.S. Population					(2) New User Sample					(3) Existing User Sample				
	Count	Mean	SD	25th Pctile	75th Pctile	Count	Mean	SD	25th Pctile	75th Pctile	Count	Mean	SD	25th Pctile	75th Pctile
ZIP: log(Total Population)	32799	7.93	1.86	6.61	9.52	12597	9.59	1.09	9.05	10.36	8206	9.93	0.92	9.56	10.53
ZIP: log(Median House. Inc.)	30891	10.91	0.39	10.67	11.13	12417	11.03	0.41	10.77	11.30	8092	11.09	0.42	10.82	11.37
ZIP: White	32799	0.83	0.21	0.77	0.97	12597	0.77	0.21	0.68	0.92	8206	0.73	0.21	0.63	0.89
ZIP: Black	32799	0.08	0.16	0.00	0.07	12597	0.11	0.17	0.01	0.13	8206	0.12	0.17	0.02	0.14
ZIP: Under 18 Year Olds	32799	0.21	0.08	0.18	0.25	12597	0.22	0.06	0.19	0.25	8206	0.22	0.06	0.19	0.25
ZIP: 18 to 24 Year Olds	32799	0.09	0.09	0.05	0.10	12597	0.10	0.11	0.07	0.10	8206	0.11	0.12	0.07	0.10
ZIP: 25 to 44 Year Olds	32799	0.23	0.08	0.19	0.27	12597	0.25	0.07	0.22	0.28	8206	0.26	0.07	0.23	0.29
ZIP: College or More	32763	0.24	0.17	0.13	0.31	12576	0.33	0.18	0.19	0.44	8192	0.37	0.19	0.22	0.49
ZIP: Poverty White	32467	0.13	0.11	0.06	0.17	12486	0.12	0.09	0.06	0.15	8133	0.12	0.09	0.06	0.15
ZIP: Poverty Black	21643	0.25	0.28	0.01	0.37	11699/	0.23	0.21	0.07	0.32	7907	0.21	0.18	0.07	0.30
ZIP: Industry Retail	32523	0.11	0.07	0.08	0.13	12584	0.11	0.04	0.09	0.13	8203	0.11	0.03	0.09	0.13
ZIP: Industry Wholesale	32523	0.02	0.03	0.01	0.03	12584	0.03	0.01	0.02	0.03	8203	0.03	0.01	0.02	0.03
ZIP: Industry Information	32523	0.02	0.03	0.00	0.02	12584	0.02	0.02	0.01	0.03	8203	0.02	0.02	0.01	0.03
County: Cases per Capita	3220	1.19	1.23	0.45	1.57	227	0.44	0.58	0.01	0.82	182	0.54	0.60	0.01	0.91
County: Social Distancing	82458	-0.14	0.21	-0.28	0.00	6156	-7.44	23.62	-3.54	0.00	5731	-18.67	57.23	-9.23	0.00

Notes.—Sources: DataCamp (*15*); American Community Survey 2014-2018 (*29*); Unacast (*31*). The table reports the number of observations, mean, standard deviation (SD), 25th percentile, and 75th percentile for all zip codes in the United States, in our new user sample, and in our existing user sample, respectively. Zipcode characteristics include: logged total population, logged median household income, the race distribution (the share White, Black), the age distribution (the share under age 18, 18-24, 25-44), the share of individuals with at least a college degree, the poverty rate for Whites and Blacks, and the share of workers in the retail trade, wholesale, and information sector. County controls include: cumulative coronavirus cases per capita and social distancing compliance from Unacast. Significance is denoted as follows: * 0.10 ** 0.05 *** 0.01.

Table S2. Heterogeneity in the Effects of Nonessential Business Closures.

	log(New user registration)			
	(1)	(2)	(3)	(4)
Nonessential Business Closures (NBC)	.31***	.37***	.42***	.30***
	[.02]	[.03]	[.02]	[.02]
High log(Median Household Income)	-.07***			
	[.02]			
NBC x High log(Median Household Income)	.10**			
	[.02]			
High Black		-0.02*		
		[.01]		
NBC x High Black		0.01		
		[.02]		
High Industry Retail			0.05***	
			[.01]	
NBC x High Industry Retail			-0.10***	
			[.01]	
High College or More				-0.07***
				[.02]
NBC x High College or More				0.11*
				[.01]
R-squared	0.25	0.25	0.25	0.25
Sample Size	109555	109555	109555	109555
Zip Code Controls	Yes	Yes	Yes	Yes
County Controls	Yes	Yes	Yes	Yes
Week Fixed Effect	Yes	Yes	Yes	Yes
State Fixed Effect	Yes	Yes	Yes	Yes

Notes.—Sources: DataCamp (*1*); American Community Survey 2014-2018 (*2*); Unacast (*3*). The table reports the coefficients associated with regressions of logged new user registrations on an indicator for whether the state has a nonessential business closure in place, conditional on controls and state and week fixed effects. Zipcode controls include: logged total population, logged median household income, the race distribution (the share White, Black), the age distribution (the share under age 18, 18-24, 25-44), the share of individuals with at least a college degree, the poverty rate for Whites and Blacks, and the share of workers in the retail trade sector. County controls include: cumulative coronavirus cases per capita and social distancing compliance from Unacast. Significance is denoted as follows: * 0.10 ** 0.05 *** 0.01.

Table S3. Heterogeneity in the Effects of Nonessential Business Closures.

	log(Exercises completed by existing users)			
	(1)	(2)	(3)	(4)
Nonessential Business Closures (NBC)	.11**	0.02	0.04	.09**
	[.04]	[.04]	[.04]	[.04]
High log(Median Household Income)	.08***			
	[.03]			
NBC x High log(Median Household Income)	-.08***			
	[.02]			
High Black		-0.06**		
		[.03]		
NBC x High Black		0.06*		
		[.03]		
High Industry Retail			-0.08***	
			[.03]	
NBC x High Industry Retail			0.04*	
			[.02]	
High College or More				-0.01
				[.03]
NBC x High College or More				-0.04
				[.03]
R-squared	0.00	0.00	0.00	0.00
Sample Size	122120	122120	122120	122120
Zip Code Controls	Yes	Yes	Yes	Yes
County Controls	Yes	Yes	Yes	Yes
Week Fixed Effect	Yes	Yes	Yes	Yes
State Fixed Effect	Yes	Yes	Yes	Yes

Notes.—Sources: DataCamp (*1*); American Community Survey 2014-2018 (*2*); Unacast (*3*). The table reports the coefficients associated with regressions of weekly exercises among existing users on an indicator for whether the state has a nonessential business closure in place, conditional on controls and state and week fixed effects. Zipcode controls include: logged total population, logged median household income, the race distribution (the share White, Black), the age distribution (the share under age 18, 18-24, 25-44), the share of individuals with at least a college degree, the poverty rate for Whites and Blacks, and the share of workers in the retail trade sector. County controls include: cumulative coronavirus cases per capita and social distancing compliance from Unacast. Significance is denoted as follows: * 0.10 ** 0.05 *** 0.01.

SI References

1. DataCamp. Available at: https://www.datacamp.com/ [Accessed November 1, 2020].
2. U.S. Census Bureau, American Community Survey (ACS) 2014–2018 (5-Year Estimates), 2019.
3. Unacast. Schema for Covid-19 social distancing scoreboard. Available at https://www.unacast.com/covid19/docs/schema-for-covid-19-social-distancing-scoreboard [Accessed November 1, 2020].
